# Imperial Health Problems

**Published:** 1911-09-30

**Authors:** 


### IMPERIAL HEALTH PROBLEMS.

That the prevention of disease should be approached not so much from the humanitarian standpoint as from the economic point of view is a sound principle. In the case of our own Empire this argument applies with great force. The amount of useless expenditure caused by tropical diseases in our colonies has been very considerable, but the money that has been spent in combating these maladies has yielded a return admitted on all sides to have been a profitable one. There remains a vast amount yet to be done, and it is to be hoped that the future ministers of the Crown will foster this most economical insurance, and will realise, as did Mr. Joseph Chamberlain, that the duty of attention to public health is an axiom of our imperial rule.

### Mr. Chamberlain and Imperial Medicine.

The great services rendered by Mr. Chamberlain to the schools of tropical medicine are well known, and to him is due the credit of having been the first minister to realise and to formulate the value of health to our imperial power. A passage in his speech at the festival dinner of the Seamen's Hospital Society in May 1899 is worth repeating. "The man," he said, "who shall grapple with this foe to humanity . . . and shall make the tropics liveable for white men, who shall reduce the risk of disease to something like an average—will do more for the world, more for the British Empire, than the man who adds a new province to the wide dominions of the Queen."

To this statesman Dr. Francis Fremantle has dedicated most appropriately his recently published work entitled "Health and Empire."* This book, we venture to say, will be accorded a large measure of popularity ; it is so pleasantly written that even as a mere traveller's tale it will meet with substantial success. The author writes for general rather than for professional consumption, and seeks to impress on the reader the fundamental importance to a modern empire of a due attention to the public health. As Dr. Fremantle remarks, the subject of public health is a "study of empires, not villages; of all climates, not one; of all races, all conditions, all times." If the microscope is essential for the scientific progress of public health, there is still more need of telescopic study in administrative methods and practical experiments. Local experience needs to be supplemented by a more cosmopolitan method.

### Faults in the Past.

The shortcomings of the past are now being slowly overtaken, and in increasing degree the attention of the Legislature and the public is being directed to the supreme importance of the popular health. "Salus populi suprema lex" is now less

* John Ouseley, Ltd., 7s. 6d. net.

of a copy-book maxim and more of a practical text. Humanity plays only a minor part in the rivalry on which prosperity of communities depends; the maximum effective output of a nation varies immediately with its health and strength, and the principles of economics call insistently for observance. In the beginning of his book Dr. Freemantle has developed a prefatory argument which lucidly outlines an offensive policy as the best defence, and points to the stern lessons of the past as evidence more than sufficient to convince anyone of the value of health to empire.

The author had certainly no reason to complain of boredom on his tour; few trips round the world could compete with his in incident and profitable interest, and he has successfully reflected this profitable interest in the well-written and virile chapters of an absorbing and purposeful book. The description of a six months' life and death struggle with the plague in the Punjab is given with a masterly vividness and should prove a good object-lesson to our critical stay-at-homes in the difficulties and magnitude of this problem—one of our greatest responsibilities of empire and one which has cost over seven million lives during the last fourteen years. Incidentally the circumstances of the unfortunate Mulkowal episode are related at some length, and its practical effects demonstrated by personal observations on the spot.

### Town-Planning.

The subject of town-planning is now beginning to receive more adequate attention, both at home and abroad, and the chapters relating the difficulties and also the successes of efforts in this direction will be read with interest. Kuála Lumpúr, the capital of the Federated Malay States, is contrasted with the town of Hong Kong, where the endeavours made to induce the Chinese to leave their slums and to inhabit better quarters in Kowloon are meeting with very moderate success. Overcrowding is the greatest possible evil in Hong Kong, and when it is realised that this spot is a regular "cooking-pot" and distributing centre of plague for the whole world, it becomes a matter for deep concern that in the new district of Kowloon, across the water, there is already serious overcrowding in existence. To attempt to follow here the author in his travels is out of the question; we can but congratulate him on the way he has drawn valuable object-lessons from his globe-trotting. Whether he is describing the realities of the Russo-Japanese war and the practical enthusiasm of the Japanese Red Cross Society, or the extraordinary health-faking and almost incredible jobbery that went on in San Francisco in 1900-02, Dr. Fremantle always succeeds in placing before the reader telling pictures, to which numerous exceedingly good photographs, taken by himself, add elucidative interest.

### The Problem of Physical Efficiency.

The book does not, however, leave untouched the consideration of the practical steps which shall tend to secure increased physical efficiency for the four hundred million inhabitants of the British Empire. In the last chapter the author points out the urgent need for the widest possible field for the study of health, and insists on the necessity for the co-ordination of the experience of health and disease in every part of the globe. To this end an efficient Intelligence Department requires to be instituted and organised to collect information of disease occurring in all parts of our dominions, and which would be able to issue official news to the Press while it was still fresh. A weekly system of notification and distribution from a single imperial centre *by wire* would save many an outbreak and much money. The formation of a permanent Imperial Advisory Council for the promotion of health is suggested, which would take over the work of the scattered advisory committees already existing. This body would appoint special committees for the consideration of special topics, and would despatch missions for local investigations of exceptional conditions. Much might have been done by such bodies during recent years had they been formed for the consideration of sleeping sickness, before two-thirds of the population of the infected district in Uganda had been killed by it; of the plague in India during the first eight years of the outbreak, when but little was done; of beri-beri; of tuberculosis on the Rand; of midwifery; of the changes required in medical education, and many other important questions having a direct bearing on the public health.

Such a body would employ for purposes of inspection and report an ever-increasing staff, experienced both in temperate and tropical sanitation from the administrative standpoint. This inspectorate, as Dr. Fremantle points out, is already to hand waiting idle in this country with nothing to do. He suggests the employment of retired officers of the Indian, Naval, Army, and Colonial medical services and laymen of administrative experience. Like the admirable inspectorate of the Local Government Board, which has acquired the respect of every district in this country, such an imperial inspectorate would earn for the advisory council proposed the confidence and support of every Government.

